# Heterosubtype Neutralizing Responses to Influenza A (H5N1) Viruses Are Mediated by Antibodies to Virus Haemagglutinin

**DOI:** 10.1371/journal.pone.0007918

**Published:** 2009-11-20

**Authors:** Jean-Michel Garcia, Stephanie Pepin, Nadège Lagarde, Edward S. K. Ma, Frederick R. Vogel, Kwok H. Chan, Susan S. S. Chiu, J. S. M. Peiris

**Affiliations:** 1 HKU-Pasteur Research Centre, Hong Kong Special Administrative Region, China; 2 Sanofi Pasteur, Marcy l'Etoile, France; 3 Department of Microbiology, University of Hong Kong, Hong Kong Special Administrative Region, China; 4 Department of Paediatrics and Adolescent Medicine, University of Hong Kong, Hong Kong Special Administrative Region, China; Instituto Butantan, Brazil

## Abstract

**Background:**

It is increasingly clear that influenza A infection induces cross-subtype neutralizing antibodies that may potentially confer protection against zoonotic infections. It is unclear whether this is mediated by antibodies to the neuraminidase (NA) or haemagglutinin (HA). We use pseudoviral particles (H5pp) coated with H5 haemagglutinin but not N1 neuraminidase to address this question. In this study, we investigate whether cross-neutralizing antibodies in persons unexposed to H5N1 is reactive to the H5 haemagglutinin.

**Methodology/Principal Findings:**

We measured H5-neutralization antibody titers pre- and post-vaccination using the H5N1 micro-neutralization test (MN) and H5pp tests in subjects given seasonal vaccines and in selected sera from European elderly volunteers in a H5N1 vaccine trial who had detectable pre-vaccination H5N1 MN antibody titers. We found detectable (titer ≥20) H5N1 neutralizing antibodies in a minority of pre-seasonal vaccine sera and evidence of a serological response to H5N1 in others after seasonal influenza vaccination. There was excellent correlation in the antibody titers between the H5N1 MN and H5pp tests. Similar correlations were found between MN and H5pp in the pre-vaccine sera from the cohort of H5N1 vaccine trial recipients.

**Conclusions/Significance:**

Heterosubtype neutralizing antibody to H5N1 in healthy volunteers unexposed to H5N1 is mediated by cross-reaction to the H5 haemagglutinin.

## Introduction

Avian influenza (A/H5N1) virus continues to be endemic in poultry flocks in many Asian and African countries. It occasionally transmits zoonotically to humans and continues to pose a pandemic threat. One of the requirements of a pandemic virus is that the human population is immunologically naive to the new pandemic haemagglutinin. While protection to influenza is believed to be subtype specific, it has been shown that exposure to one subtype of influenza A can induce immunity that is cross-protective against other subtypes [1]–[6]. Such broad immune protection is termed “heterosubtypic immunity” (HSI) and while it may not provide sterilizing immunity it may reduce morbidity and mortality. In the context of pandemic emergence, such heterosubtypic immunity could confer some level of population immunity and may even prevent some avian influenza virus subtypes from becoming pandemic viruses, thus providing an additional barrier to inter-species transmission.

There is some evidence for HSI in humans. Recent influenza A infection seemed to confer partial protection against symptomatic disease during the H2N2 pandemic when the pandemic strain did not share either the HA or NA with the preceding seasonal influenza viruses [7]. More recently, a retrospective study of the archived records of laboratory-confirmed cases of influenza before and during H2N2 pandemic of 1957 also concluded that those who had been symptomatic during previous influenza season(s) had accumulated (age dependent) heterosubtypic immunity reducing attack rate with the pandemic subtype [8]. In general, such heterosubtypic cross protection is largely believed to be mediated by cross reactive cell mediated immunity [9]. However there has also been some suggestion of heterosubtype protection by neutralizing antibody, at least via antibodies to the NA [10].

Cross-neutralizing antibodies are also relevant in interpreting sero-epidemiological studies of human infections with avian influenza viruses such as H5N1 and H9N2 [11]. Approximately 3% of healthy adult US volunteers in H5N1 vaccine trials had evidence of antibody to H5N1 virus in their pre-vaccine sera detected in microneutralization and horse erythrocyte haemagglutination inhibition tests [12]. These antibodies were presumed to be heterosubtypic antibodies since these volunteers were unlikely to have been naturally exposed to H5-subtype viruses. Similarly, 24 of 60 volunteers in a H9N2 vaccine clinical trial in the UK had neutralising antibody to H9N2 virus prior to being vaccinated [11]. The seropositive persons were all UK-residents born before 1969 and it was hypothesised that prior natural exposure to the H2N2 virus subtype may be responsible for some of these cross reactions. Using an H9N1 reassortant virus, they demonstrated that the neutralizing activity was directed to the H9-hemagglutinin rather than the N2 neuraminidase. Finally, recent publications demonstrated the existence of cross-subtype neutralizing antibodies [13] directed against a conserved domain of haemagglutinin that acts by blocking the conformational rearrangement of HA2 sub-domain in the fusion step of viral entry [14], [15].

We have developed, optimised and validated a H5 pseudoparticle-based (H5pp) serological assay for the identification of H5N1 neutralizing antibodies and this assay correlates well with the conventional micro-neutralization test [16]. As these H5pp only contain the virus HA, this allows us an opportunity to investigate neutralizing antibody to the virus HA alone, avoiding the confounding antibody responses to the NA.

## Materials and Methods

### Serum samples

Pre and post seasonal influenza vaccine sera from 98 children who received the Fluarix, GlaxoSmithKline Biologicals, Belgium containing influenza A/New Caledonia/20/99 (H1N1)-like, A/California/7/04 (H3N2)-like and B/Shanghai/361/02-like virus antigens in the winter of 2005 were available from previous studies on seasonal influenza vaccination [17]. Similar pre and post vaccine sera from a cohort of community dwelling elderly (n = 118) given the influenza inactivated split-virion influenza vaccine Vaxigrip (sanofi pasteur, France) during the winter of 2003 was also used in this study. The vaccine contained the antigens A/Moscow/10/99 (H3N2); A/New Caledonia/20/99 (H1N1) and B/Hong Kong/330/2001 [18]. Sanofi pasteur (Swiftwater, PA, USA) provided a panel of 20 pairs of pre and post vaccination serum samples from adults vaccinated with seasonal influenza vaccines containing A/New Caledonia/20/99 (H1N1), A/Wisconsin/67/05 (H3N2), and B/Malaysia/2506/04). They provided pre and post vaccination sera from volunteers vaccinated with H5N1 vaccines prepared using A/Vietnam/1194/2004 NIBRG-14 (H5N1) (35 pairs) or A/Vietnam/1203/2004 seed strains (7 pairs). These served as the “positive control” group of sera. Finally, sanofi pasteur provided us sera from 16 selected volunteers aged ≥60 years from a European H5N1 vaccine clinical trial who had detectable H5N1 MN antibodies (geometric mean of the two runs measured ≥20 (1/dilution)) to A/Vietnam/1194/2004 NIBRG-14 strain prior to vaccination. Ten elderly random subjects from the same vaccine trial who did not have antibody titer against A/Vietnam/1194/2004 NIBRG-14 strain at D0 (two runs measured at D0 titer of <20) were included as controls (ClinicalTrials.gov NCT00415129). Post vaccine sera were collected at 21 days post vaccination for children and adults; and 4 weeks post vaccination for elderly.

### H5pp assay

H5 haemagglutinin (A/Cambodia/408008/05; clade 1) pseudotyped lentiviral particles (H5pp) were produced by co-transfection in HEK-293 cells of plasmids coding for the H5 surface envelope as well as the HIV-1 backbone and the luciferase reporter gene as described previously [19]. The experimental parameters affecting the H5pp assay and its clinical validation in a panel of sera from patients with H5N1 disease and controls have been previously reported [16]. The H5pp assay was carried out as follows. MDCK cells (4000 cells/well) were seeded in white 96-well plates (Perkin Elmer) in 50 µL of DMEM (GIBCO) complemented with 2.5% foetal bovine serum (Invitrogen) and 1% Penicillin-Streptomycin (Invitrogen) (thereafter called “complete medium”). The next day, H5pp were incubated with two-fold serial dilutions of serum (starting dilution 1∶20, 60 µL/well total) for 2h at 37°C (5% CO_2_ incubator). Subsequently, 100 µL of fresh complete medium was added, mixed and 140 µL of the mix was transferred back to the wells after discarding the old medium. After 48h incubation at 37°C in a 5% CO_2_ incubator, 100 µL of Steady-Glo (Promega) luciferase substrate was added. Luminescence was read 15 minutes later using a Micro-beta (Perkin Elmer) plate reader. The neutralization titer was defined as the reciprocal of the serum dilution that neutralized 50% of signal computed by fitting the 12 dilutions points with the Hill model [20].

### Microneutralization test

The micro-neutralization test (HKU-MN) at the HKU laboratory was done as described previously [21] for the paediatric and elderly cohort who received the seasonal influenza vaccine. One hundred tissue culture infectious dose 50 (100 TCID_50_) of A/Vietnam/1194/04 (H5N1) virus was mixed with an equal volume of serial dilutions of serum in quadruplicate, incubated for 1 hour at 37°C and the virus antibody mixture was added to a preformed monolayer of MDCK cells. The plates were incubated for 3 days and the cytopathic effect was visually assessed using an inverted microscope. Virus back titrations were included to confirm the challenge dose was as expected. The highest serum dilution protecting more than half of the wells was taken as the antibody titer. This method is used for all the results presented in the paper with the exception of elderly sera from sanofi pasteur vaccination tested as below.

An alternative microneutralization method (SP-MN) was used in the sanofi pasteur laboratory for the 26 pairs of sera from sanofi pasteur H5N1 vaccine trial as described previously [22]. Serum samples were tested for neutralizing activity against the NIBRG14 (A/Vietnam/1203/2004) (H5N1) reassortant vaccine strain virus [23]. The key difference in the latter method was that evidence of virus neutralization was detected by ELISA in virally infected cells using antibodies to the virus nucleoprotein rather than by observing viral cytopathic effect. We had previously established that there was excellent correlation in the antibody titers obtained by the two MN assays above (unpublished data).

### Statistical analysis

In both MN and H5pp tests we have used an antibody titer of ≥20 as a positive cut-off for the purpose of these analyses and negative sera (titers <20) are scored as 10 for the purpose of computation. Titers of 80 are generally regarded as a cut-off for diagnostic purposes, based on the discrimination between H5N1 infected patients and controls [24]. However, our purpose here is not diagnostics or sero-epidemiology but to understand heterosubtypic antibody responses and we consider titers between 20 and 80 to be very relevant in that regard. From a diagnostic point of view, a positivity cut-off of >20 has similar significance for H5pp assay as for the MN (data not shown; [16]).

Two-sample unequal variance one-tailed distribution Student's t-test was used to assess the differences in geometric titers with significance level α = 0.05.

### Ethics statement

All the sera mentioned in this work were provided from past vaccination trials. Written informed consent were obtained prior to the start of the trials (consent from parents and the older children before enrolment for paediatric sera or the subject themselves for adults). Study protocols were approved by the joint institutional review board of the University of Hong Kong and Queen Mary Hospital (Hong Kong) for the paediatrics sera [17]; by the Institutional Review Board of the University of Hong Kong and Hospital Authority Hong Kong West Cluster for the elderly sera [18] and by the UK and Belgium IECs (National Research Ethics Service NHS, Oxfordshire REC B and Ethics Committee of the University Hospital of Antwerp, respectively) for adults sera (ClinicalTrials.gov NCT00415129) as detailed in cited references. All studies complied with the Declaration of Helsinki of 1975, as revised in 1983.

## Results

Neutralizing antibodies titers were measured in parallel by classical micro-neutralization (MN) and by pseudoparticle-based neutralization (H5pp) assays for pre and post seasonal influenza vaccination sera from 98 children, 20 adults and 118 elderly (≥60 years old). The antibody responses to seasonal influenza viruses H1N1, H3N2 and influenza B in these sera have been previously reported [17], [18]. As expected, most subjects were negative (titer <20) for H5 antibody in both MN and H5pp assays in both pre and post vaccine sera. Of 118 pre-vaccine sera from subjects aged ≥60 years, one (0.8%) had H5-neutralizing antibody in the MN test, while 8 (6.8%) sera (including the serum with MN antibody) were sero-positive in the H5pp tests (Table 1). Six additional individuals sero-converted by the H5pp test after seasonal influenza vaccination. Of the pre-vaccination sera of 98 children, none were H5 antibody positive by MN and 1 (1%) was positive by the H5pp test. Two and 6 children sero-converted respectively by the MN and H5pp test post vaccination (Table 1).

**Table 1:** Effect of seasonal vaccination on H5 neutralizing antibody titers measured by the microneutralization (MN) and the H5pp tests in paired sera from children, adults, and elderly after vaccination with seasonal influenza vaccine and H5N1 vaccine.

Type of vaccine	Subjects	N^c^	Sera with titer ≥20				Geometric mean titer^d^					
			H5pp		MN		H5pp			MN		
			pre	post	pre	post	pre	post	p-value	pre	post	p-value
Seasonal	Children a	98	1	7	0	2	10.2	11.6	0.023	10.0	10.5	0.252
	Adult b	20	1	2	0	0	10.4	11.9	0.196	10.0	10.0	-
	Elderly a	118	8	14	1	2	11.5	12.9	0.045	10.1	10.2	0.319
H5N1	Adult b	42	1	39	1	34	10.7	237.6	<0.001	10.4	41.8	<0.001

a Patient cohorts from Hong Kong.

b Patient cohorts from Europe.

c N: Number of paired samples.

d For the computation of geometric mean titers, negative sera (titer <20) are assigned value of 10 (first dilution tested 20). Student's t-test was used to calculate p-value.

**Figure 1:**
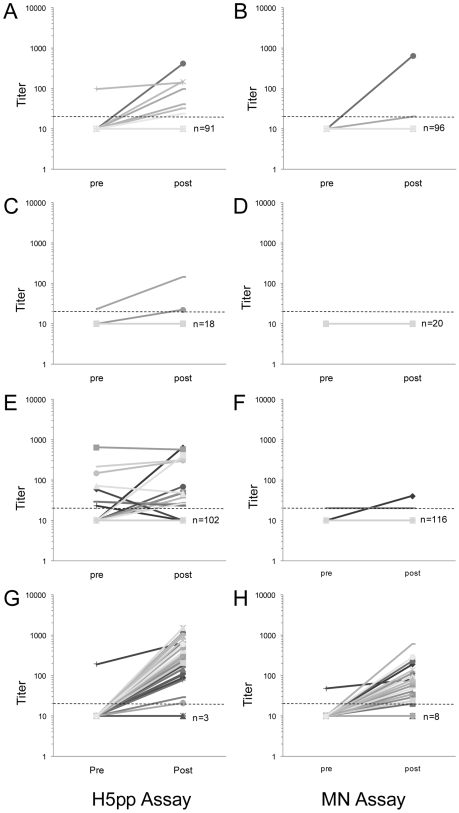
Comparison of H5 neutralizing antibodies tested by MN and H5pp tests for different age groups. Titers were measured from paired sera of 98 children (A, B), 20 adults (C, D) and 118 elderly (E, F) given seasonal influenza vaccination; (G, H) 42 pairs sera from adults recipient of H5N1 clinical candidate vaccines. Dashed line marks the positivity criteria (titer≥20). Each line represents a paired serum from one individual. Where more than one person has identical pre and post antibody titers, the number of persons with these overlapping results is denoted as n. The MN titers were determined at HKU using the MN-HKU method (see methods).

The pre-vaccine geometric mean H5pp antibody titers (GMT) for each group were 10.2 for children, 10.4 for adults and 11.5 in the elderly. The GMT of pre-vaccine sera measured with MN were no different between the different age groups (p>0.1). There was a discernible H5pp antibody response to seasonal influenza vaccine in a few children and elderly subjects. We could measure a significant increase of GMT after vaccination both in children (GMT pre vaccination 10.2 versus 11.6 post, p = 0.023) as well as in elderly (GMT 11.5 pre versus 12.9 post vaccination, p = 0.045) as shown in Figure 1 and Table 1. Such a response was less obvious in the MN test and not statistically significant (p>0.2). In contrast and as expected, there was a marked increase in GMT post-vaccination with H5N1 clinical candidate vaccines by both H5pp and MN assays (p<0.001) (Figure 1G and 1H, Table 1).

**Figure 2:**
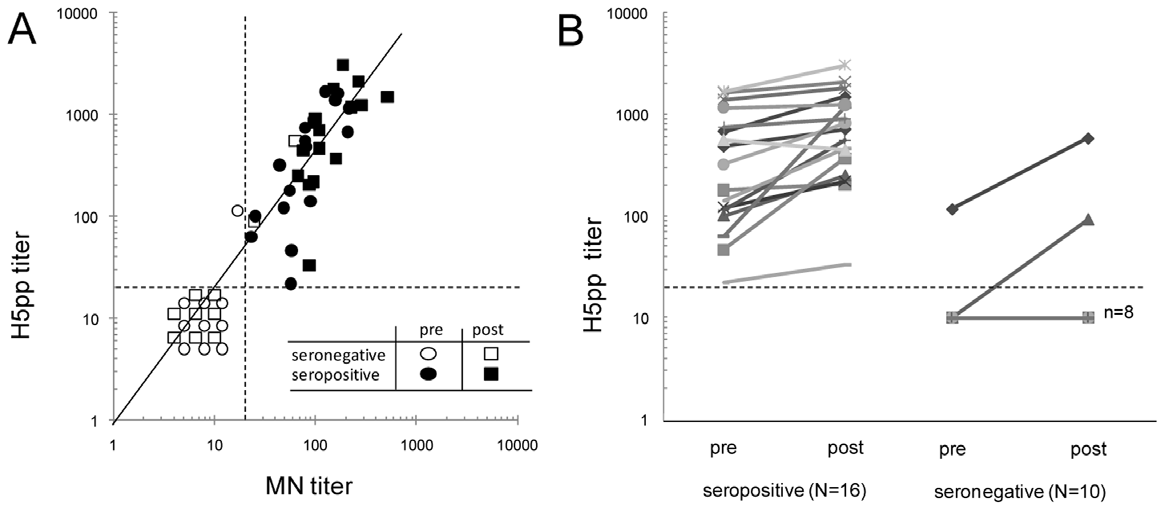
Comparison of titers obtained with H5pp versus MN assays for two panels of paired sera. Titers (reciprocal dilution of serum that gives 50% neutralization) were determined for sera from elderly (>65 yr) recipient of H5N1 vaccine selected to include 16 with pre-vaccination MN H5N1 titer ≥20 and 10 randomly selected seronegative persons with MN titer <20 and HI <8. The post vaccination was sampled 21 days post vaccination. The graph (A) shows the correlation of antibody titers in pre- (circles) and post (square) vaccination sera obtained with the H5 MN (sanofi pasteur method) and H5pp tests for those who were MN seropositive (black markers) and seronegative (open markers) pre-vaccination. Graph (B) shows the pre- and post H5N1 vaccination H5pp antibody titers in those with (“seropositive”) and without (“seronegative”) pre-vaccine antibody. For computation, the sera with undetectable antibody titers were given the value of 10. Positivity criterion (titer≥20) is represented by dashed lines.

We next investigated sera collected from European volunteers above 60 years of age participating in an H5N1 vaccine trial. We chose 16 subjects who were found to be H5N1 MN antibody positive pre-vaccination and 10 others who were MN (and HI) negative (<20) as controls. All 16 volunteers who prior to vaccination were MN antibody positive were H5pp positive. One of 10 who was MN negative (MN titer 17) was H5pp-antibody positive (≥20). There was a good linear correlation between the H5N1 MN and H5pp antibody titers in these sera (Figure 2A) indicating that the MN H5-antibody titers were largely explainable by heterosubtypic cross-reactions to the virus HA. The geometric mean H5pp antibody titer of those 16 individuals with pre-existing H5 antibody increased from 279.7 to 605.2 (p = 0.038); and from 12.7 to 18.6 (not statistically significant, p>0.2) in the 10 individuals who were H5-seronegative pre-vaccination. The serological response to the H5N1 vaccine is shown in Figure 2B.

## Discussion

Prior to immunization, none of 98 children and 1 of 118 (0.8%) elderly individuals had detectable MN H5-antibody titers while 1/98 (1%) children and 8/118 (6.8%) elderly persons were positive in the H5pp test which is known to be a more sensitive assay for antibody detection [16]. All sera positive in the MN test were also positive in the H5pp test demonstrating that presumed H5-MN neutralising activity in human sera is strongly associated with cross reactivity to the viral HA. None of these persons had known H5N1 disease but since this study cohort were long term residents in Hong Kong, inadvertent exposure to H5N1 infected poultry markets or wild birds, though unlikely, cannot be excluded. We therefore also tested a cohort of 62 adults volunteers from a seasonal influenza (n = 20) and H5N1 vaccine (n = 42) studies in Europe where inadvertent exposure to H5N1 virus is highly improbable. Prior to vaccination, one of these volunteers had detectable H5 antibody by MN and two were positive for H5pp antibody (Table 1).

To further investigate these observations on heterosubtypic neutralizing antibodies, we used selected sera from persons over 60 years of age recruited to a European H5N1 vaccine trial. We selected 16 individuals whose pre-vaccine sera had detectable (titers≥20) H5N1 MN antibody and 10 persons who did not as controls. All of the MN positive sera were also positive in the H5pp test and the titers in the two assays were highly correlated (Figure 2A, Pearson ρ = 0.734, p<0.001 with all 26 pairs). Only 1 of 10 of the controls (MN antibody negative) was seropositive in the H5pp assay.

Seasonal influenza vaccine appears to induce heterosubtypic antibody responses in a minority of individuals by the MN test. In the Hong Kong vaccine cohorts 2 children (2.0%) and 1 (0.8%) person in the ≥60 years cohort had increase in H5 neutralizing antibodies by MN (Figure 1B and 1F). This was even more notable in the H5pp test with 6 children (6.1%) and 14 persons ≥60 years (5.1%) had H5 antibody responses (Figure 1A and 1E). Again all the MN sero-conversions are also reflected in the H5pp assay. As expected, most of the adults given the H5N1 vaccine sero-converted to H5N1 virus by both MN and H5pp tests (Table 1; Figure 1G and 1H).

There has been much debate on the mechanisms underlying heterosubtype cross protection and some have postulated that this is largely contributed by the virus NA [10], [25]. Indeed, DNA vaccination of mice with the H5N1 virus NA has led to cross protection against live H5N1 virus challenge. On the other hand, monoclonal antibodies to HA epitopes that cross neutralise across subtypes (e.g. H1, H2, H5, H6, H9) have been demonstrated on the HA and such antibodies cross-protect against H5N1 virus challenge in mice [13], [26]. Our data strongly argue that there are heterosubtypic neutralising epitopes within the virus HA. Since the H5pp assay only contains the virus haemagglutinin without the virus NA or M2 proteins, one can conclude that these H5pp heterosubtypic cross-reactions are mediated by antibody responses to epitopes expressed on the virus HA. Published data showed that such anti-HA heterosubtypic antibodies could be generated in vitro [15], [27]. We showed here they can be detected also in vivo. Why these cross-subtypic reactions are only manifested by a minority of individuals is unclear. Possibly the host histocompatibility antigens or other host factors (such as VHI69 gene [28]) may contribute to these unusual heterosubtypic responses.

These findings raise important questions of clinical relevance. While it is unclear whether these low H5pp antibody titers of 10–20 have any protective effect, such an effect cannot be ruled out. A single individual responded with H5N1 MN titers of ≥256 after seasonal influenza vaccination suggests that in some occasions, these heterosubtypic antibodies are within what is accepted to be within the protective range. More recently, evidence of heterosubtypic immunity has been reported [29]. If such heterosubtypic immunity induced by natural infection with seasonal influenza viruses or vaccination with seasonal influenza vaccines provides some cross immunity to H5N1, it may explain a number of puzzling observations we observe in the epidemiology of H5N1 disease. These include the observation that most people who are exposed to H5N1 virus fail to get infected by the virus [30], and also that H5N1 incidence and mortality appears to decrease in those aged 45 years or older [31]. One may hypothesize that repeated influenza infection or vaccination leads to a progressive increase of heterosubtypic immunity that protects humans from infection with H5N1 infection when they are exposed to it.

Our findings also have implications for sero-diagnosis. While many of the cross reactive H5 antibody responses we observed were below the titer of 80 which is generally regarded as a minimum neutralizing antibody titer to indicate evidence of recent H5N1 infection, some individuals clearly respond to seasonal influenza vaccine with substantially higher H5N1 neutralizing antibody titers. Thus we have to be cognizant of this fact when interpreting sero-epidemiological data. Although we have used sera with titers as low as 20 for our analysis, this does not imply that such titers can be taken as conclusive evidence of exposure to H5N1 viruses. In fact, our data suggest that low titer H5N1 neutralizing antibodies can very well arise as heterosubtypic responses to seasonal influenza viruses.

To conclude, using H5pp assay which is more sensitive and carries the H5 HA without the N1 neuraminidase on the viral particle, we demonstrated that antibodies directed to haemagglutinin can induce heterosubtypic response to influenza A.
